# Four decades of tinnitus pathology research: a bibliometric analysis of scientific trends, collaborations, and research hotspots

**DOI:** 10.1186/s12245-026-01311-0

**Published:** 2026-08-03

**Authors:** Selena M. Shen, Rakin Haq, Latha Ganti

**Affiliations:** 1 Pine View School, Sarasota, FL USA; 2 Brown University, Providence, RI USA; 3 Warren Alpert Medical School of Brown University, 222 Richmond St, Providence, RI 02903 USA

**Keywords:** Tinnitus, Bibliometric analysis

## Abstract

**Introduction:**

Tinnitus is a debilitating auditory disorder affecting approximately 14% of adults worldwide, often co-occurring with hearing loss, sleep disturbance, anxiety and depression. Despite its high global prevalence and substantial socioeconomic burden, tinnitus research remains underfunded, and effective treatments are limited due to the condition’s complex and heterogeneous pathophysiology. A comprehensive understanding of the research landscape in tinnitus pathology is essential for guiding future scientific and clinical efforts, including the evaluation of patients presenting with tinnitus in emergency and acute care settings.

**Methods:**

A bibliometric analysis was conducted on648 documents related to tinnitus pathology indexed in the Web of Science Core Collection (1977–2025). The analytical tools VOSviewer, R-bibliometrix, and Microsoft Excel were used to assess publication trends, source journals, contributing countries and institutions, author networks, and thematic keyword evolution.

**Results:**

Findings reveal a steady increase in both publication count and academic attention to tinnitus pathology, with even more rapid growth projected. The United States leads in publication volume and international collaboration, although countries like Finland and Switzerland demonstrate higher average citation impact. Authors Dirk de Ridder and James A. Henry were identified as key points of dissemination of academia. Keyword analysis revealed a historical shift from peripheral auditory models to central neurophysiological frameworks, with recent emphasis on intervention-focused topics. Thematic mapping identified “pathology,” “prevalence,” and “diagnosis as central yet underdeveloped areas requiring further academic investment.

**Conclusion:**

This bibliometric analysis contributes a comprehensive overview of the research landscape for tinnitus pathology. Current research focuses on translating our basic understanding of tinnitus into intervention. Despite recent advancements, critical knowledge gaps persist in tinnitus pathophysiology, diagnostic specificity, and prevalence metrics standardization. These findings have important implications for emergency medicine, where tinnitus may represent either a benign symptom or the manifestation of serious neurologic, vascular, or otologic disease requiring urgent evaluation.

## A bibliometric analysis of 48 years of tinnitus pathology research

### Introduction & background

**Fig. 1:**
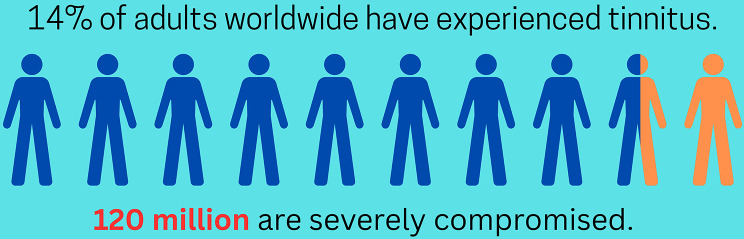
Infographic depicting tinnitus prevalence

Tinnitus is a phantom auditory sensation experienced without external sound stimulus [1]. Approximately 14% of adults worldwide experience tinnitus, with over 120 million people severely compromised [2] (Fig. 1). This risk increases significantly with age, which is particularly alarming in light of the global trend toward an aging population [3]. Far more than just a “ringing” in the ears, tinnitus often presents alongside irritability, hyperacusis, hearing loss, sleep disorders, anxiety, and depression, leading to a vicious deterioration in quality of life [4, 5]. Despite being historically underfunded [6], tinnitus still accounts for considerable financial costs for both healthcare systems and society at large [7]. Tinnitus imposes on each patient every year $1780 - $3952 for patient and family costs and $2957-$4267 indirectly (including productivity loss) [7].

A wide range of options is available for the treatment of tinnitus, including pharmacological interventions [8–12], sound-based therapies [13–24], psychological counseling [25, 26], and multimodal approaches [27–30]. In general, treatments aim to either reduce tinnitus perception or the associated emotional and psychological distress [26]. However, even the most effective current treatments are only able to reduce symptom severity rather than addressing the tinnitus precept.

Tinnitus remains incurable due to its enigmatic and highly heterogeneous pathophysiology. This lack of clarity has sparked the development of multiple tinnitus models. A widely accepted explanation is the central gain model: sensory deprivation of the auditory system - whether from age or noise-exposure induced hearing loss prompts the brain to compensate by increasing neural sensitivity and over-representing auditory input in a nonlinear manner [14, 30–32]. Other models include the asynchrony theory [14, 33], which focuses on disorganized neural timing and connectivity rather than simply excess or reduced activity, and the Jastreboff model [34, 35], which highlights the role of emotional and attentional circuits in tinnitus persistence.

Understanding tinnitus pathology is essential for developing unified, effective treatments. However, despite its importance, no research to date has offered a comprehensive overview of the landscape, trends, and emerging hotspots in this area.

A bibliometric analysis is a quantitative research method that applies mathematical and statistical techniques to evaluate patterns within academic literature, providing a comprehensive understanding of a field’s structure, trends, and knowledge gaps [36]. By analyzing bibliographic data, keyword prevalence, author productivity and collaboration, bibliometric analyses help researchers and policy-makers identify emerging topics, collaboration networks, and understudied areas [36]. Ye et al. assessed global trends in tinnitus treatment research in the 21st century and commented on the abundance of treatment options, but no successful options for remission have been identified due to tinnitus pathogenesis remaining unclear [37]. Additional studies by Hu et al. and He et al. delve into narrower treatment modalities such as mobile health interventions and cognitive behavioral therapy, respectively [26, 38]. Though these studies have illuminated promising treatment avenues, the field still lacks a systematic understanding of tinnitus pathology, which is essential to further develop these treatment strategies.

To address this crucial gap, this study leverages rigorous knowledge mapping and data visualization techniques in order to generate fresh insights into the landscape and hotspots of tinnitus pathology research. It is hoped that this research will contribute to a deeper understanding of tinnitus and, ultimately, support researchers and clinicians in advancing tinnitus management.

Although tinnitus is most commonly managed in outpatient otolaryngology and audiology settings, it is also a frequent reason for emergency department visits, particularly when symptoms present acutely, are unilateral, pulsatile, associated with sudden hearing loss, vertigo, neurologic deficits, severe anxiety, or significant sleep disturbance. Emergency physicians are often tasked with distinguishing benign tinnitus from potentially serious underlying conditions, including cerebrovascular disease, vascular malformations, intracranial pathology, medication toxicity, and acute otologic emergencies. Given the substantial healthcare utilization associated with tinnitus and the increasing emphasis on timely diagnosis and risk stratification, a comprehensive understanding of the evolution of tinnitus pathology research is highly relevant to emergency medicine clinicians. Mapping the scientific landscape of tinnitus pathology may help identify emerging diagnostic paradigms, knowledge gaps, and future research priorities that can ultimately improve the evaluation and management of patients presenting with tinnitus across acute care settings.

## Methods

**Fig. 2:**
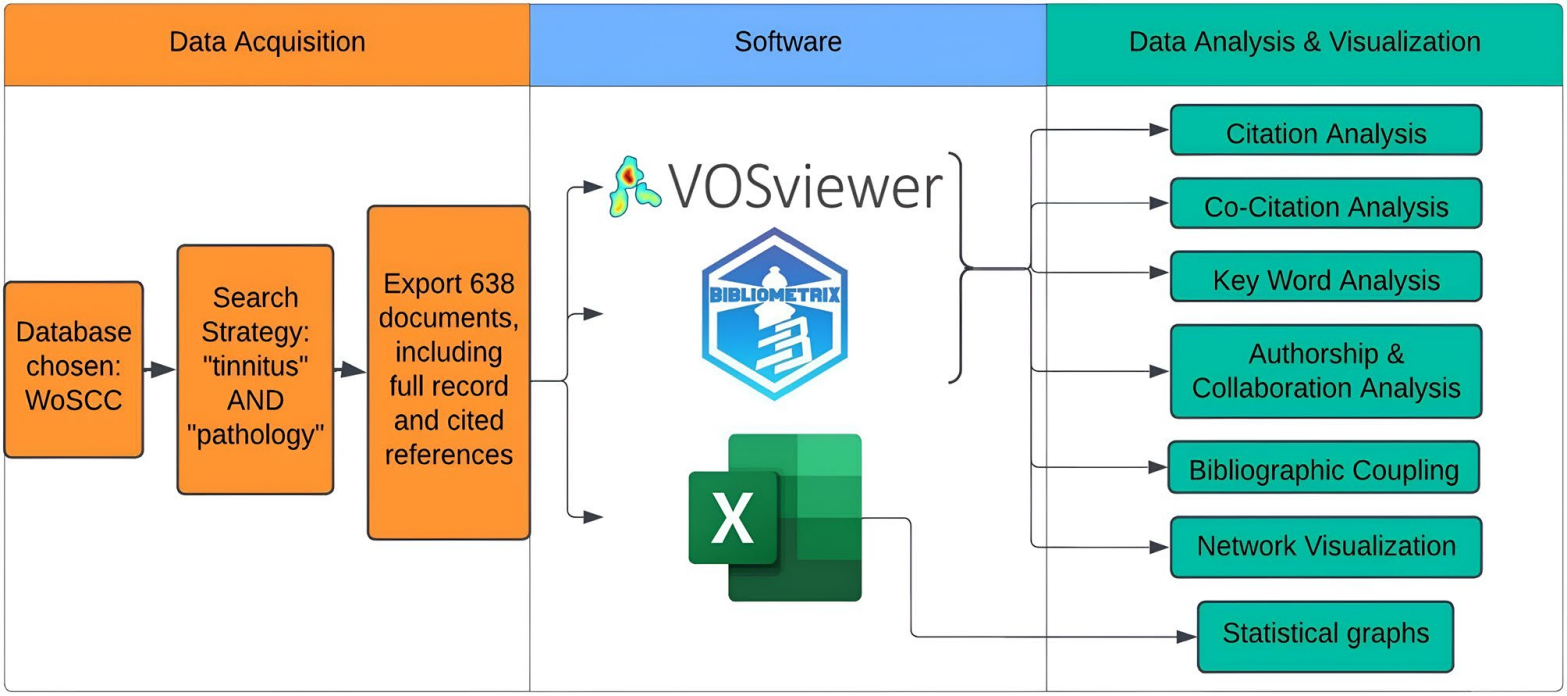
Workflow of assessing tinnitus pathology research through bibliometric analysis

Data was obtained from the Web of Science Core Collection (WoSCC). The WoSCC is considered the gold standard for bibliometric studies due to its comprehensive citation data and high-quality multidisciplinary literature resources [36, 39]. The key search items were “tinnitus” and “pathology.” A total of 638 documents relevant to the research theme of tinnitus and pathology were extracted (Table 1). The present study utilized VOSviewer and R-bibliometrix to analyze the relevant literature and visualize the results (e.g. bibliometric maps). Microsoft Office Excel 2016 was used to create statistical graphs. This process is illustrated in Fig. 2.

**Table 1:** Data description

Description	Results
Timespan	1977:2025
Sources (Journals, Books, etc.)	310
Documents	638
Annual Growth Rate %	7.11
Document Average Age	9.94
Average citations per doc	23.19
References	20,385
Document contents	
Keywords Plus (ID)	1684
Author’s Keywords (DE)	1744
Authors	
Authors	3021
Authors of single-authored docs	33
Authors collaboration	
Single-authored docs	38
Co-Authors per Doc	5.27
International co-authorships %	19.59
Document types	
article	492
article; book chapter	6
article; early access	2
article; proceedings paper	17
editorial material	10
letter	1
note	2
proceedings paper	9
review	96
review; book chapter	3

## Results

### Analysis of publication trends

**Fig. 3:**
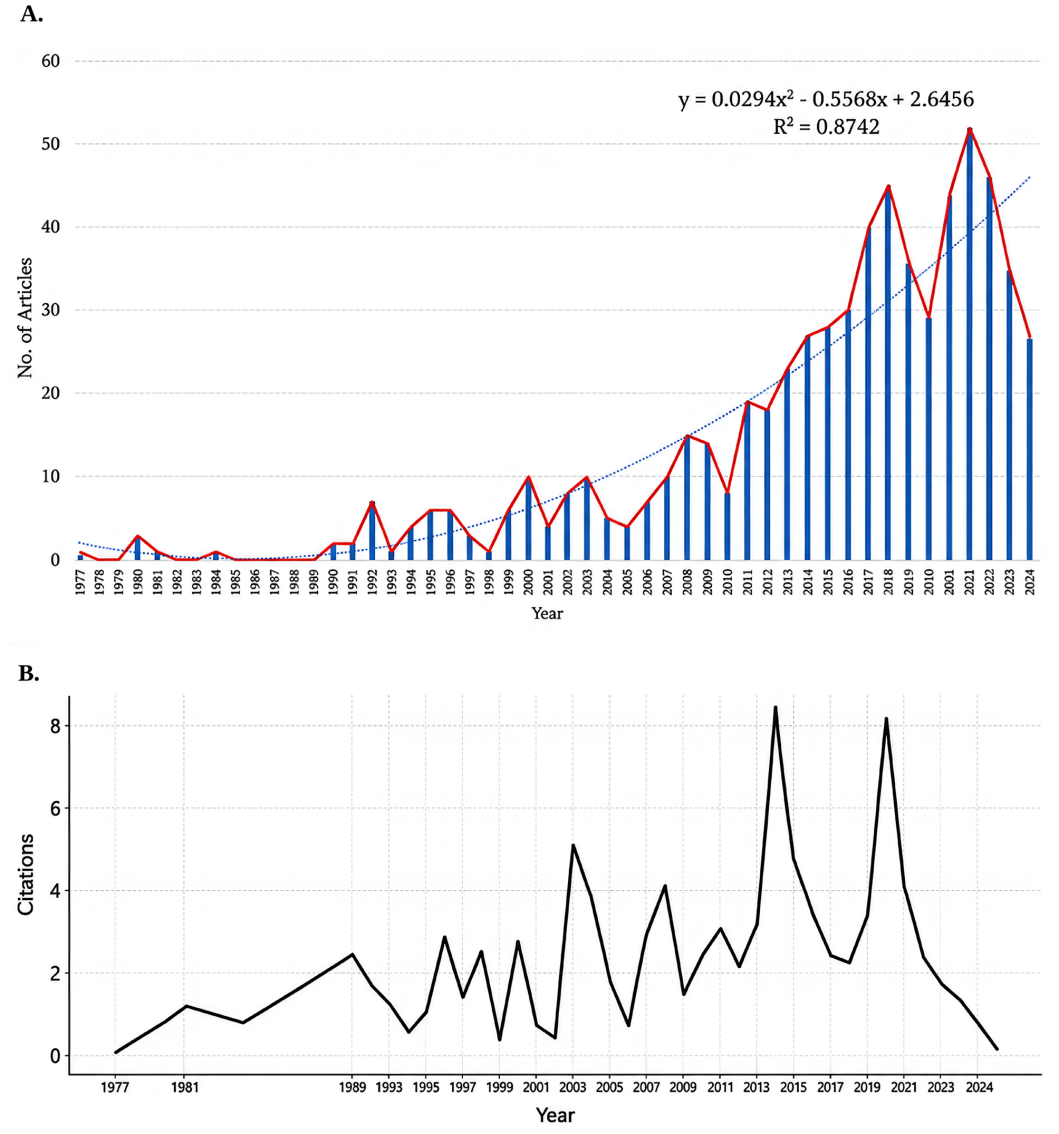
Growth trends in tinnitus pathology research from 1997–2025. (**A**) Number of publications per year. (**B**) Average citations per year

The number of publications in a field is directly linked to the probability that a publication is in the global xth percentile [40]. The average number of citations for articles is often used to determine the relative importance of a field under the assumption that publications with a greater number of citations have a larger impact [41]. Overall, the number of publications and average citations are effective indicators of the level of academic attention dedicated to a field. Plotting these two metrics over time (1977–2025) allows for the prediction of future trends. As depicted in Fig. 3A, the regression line of number of annual publications is a polynomial to the second power with an R-squared of 0.8397, indicating the number of annual publications is predicted to increase even more steeply in the future. Figure 3B shows the drastic fluctuations of average citation counts. There appears to be a general positive trend in average citations, however it must be noted that this trend may be subject to temporal bias of citation inflation [42]. There are two sharp peaks in the number of publications (2018 and 2022) and in the average number of citations (2013 and 2019).

### Analysis of journals

**Fig. 4:**
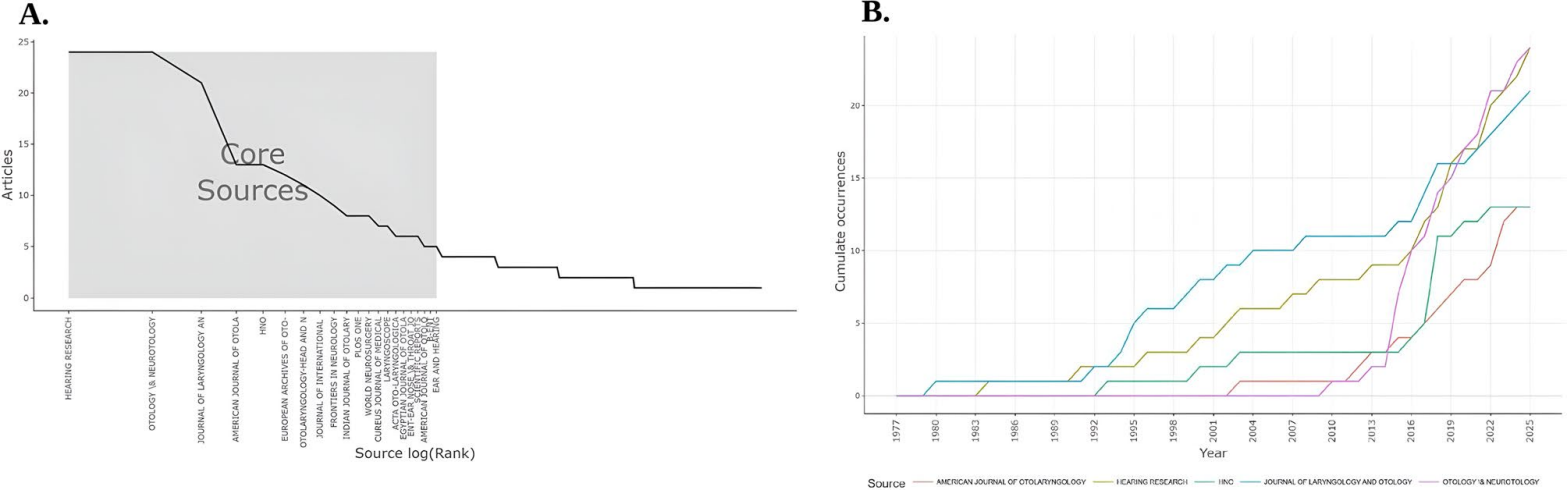
Analysis of sources. (**A**) Bradford’s law. The shaded gray area illustrates the “core” journals of the field. (**B**) Line graph of cumulative number of publications of the top 5 journals over 1977–2025

Utilizing Bradford’s law, which analyzes the scatter of citations in a field, 21 core journals in the field of tinnitus pathology were identified [43]. As illustrated in Fig. 4A, Hearing Research (24 publications, IF: 2.5, JCR: Q1) is the leading journal in tinnitus pathology research, followed by Otology & Neurotology (24 publications, IF: 1.9, JCR: Q1), and Journal of Laryngology & Otology (21 publications, IF: 1.3, JCR: Q3). These rankings were established around 2019, as reflected in Fig. 4B. Additionally, there appears to have been a sudden increase in publications among all journals around 2014.

### Countries

**Fig. 5:**
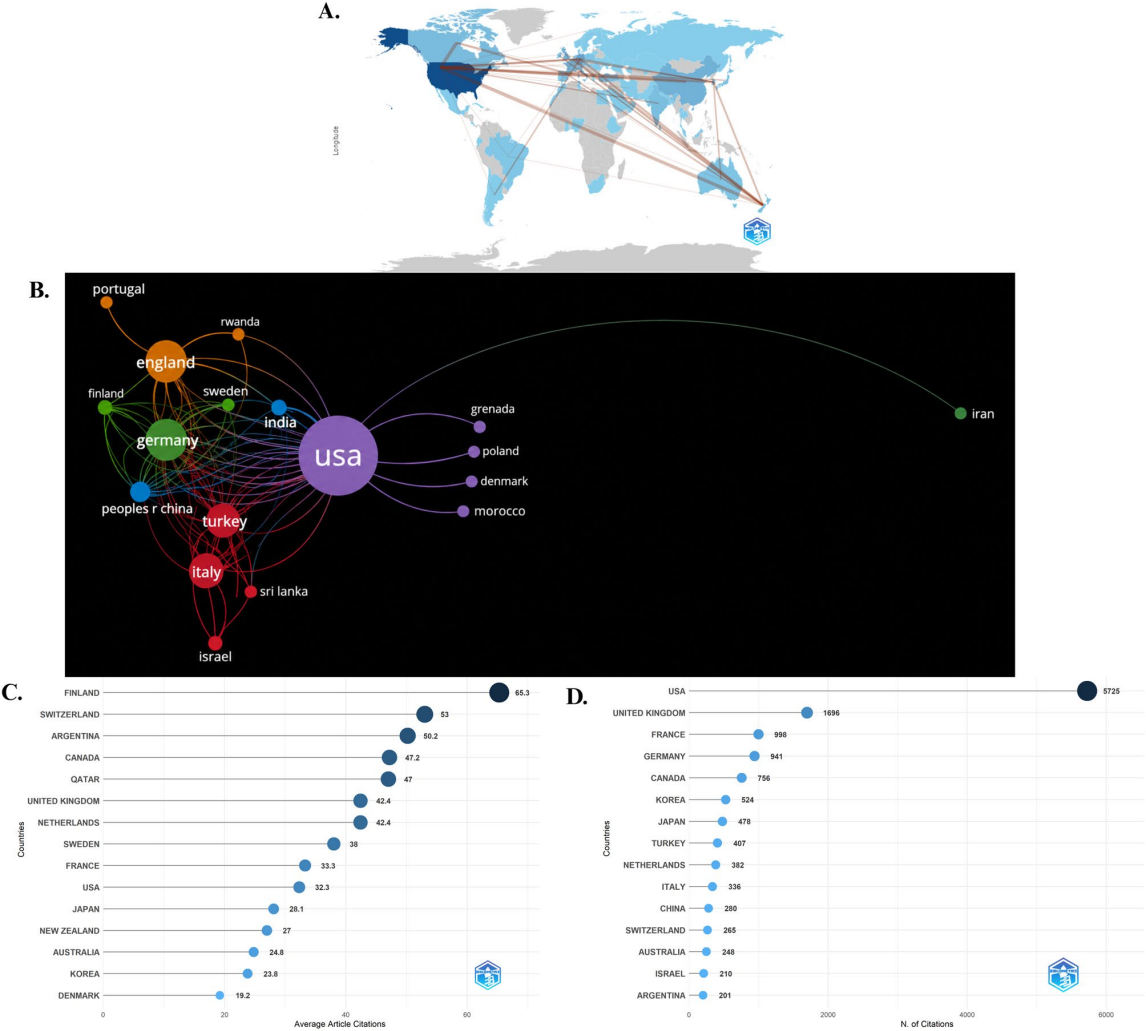
Analysis of tinnitus pathology research across countries. (**A**) Geographic map of countries. The darkness of the color represents the number of national publications, and the thickness of lines represents strength of cooperation. (**B**) Network analysis of citations among countries. The size of the node corresponds to the number of citations. (**C**) Average article citations by country. **D**) Total number of citations by country

A total of 73 countries have contributed to research on tinnitus pathology. As illustrated in Fig. 5A and C, the United States demonstrates clear dominance in both research output and international collaboration, followed by the United Kingdom, France, and Germany. The strongest international collaborative links are observed between the USA and China, as well as the USA and New Zealand (Fig. 5A). In contrast, countries such as Grenada, Poland, Denmark, Morocco, and notably Iran exhibit minimal to no international collaboration in this field (Fig. 5B). Interestingly, the countries with the highest average citations per article (Fig. 5C) differ starkly from those with the highest total citations of all publications (Fig. 5D). Although the USA ranks first in total citations (5725), significantly ahead of the UK (1696), it ranks only tenth in greatest average citations. Finland is the leading country in this metric, followed by Switzerland and Argentina, each with a relatively modest number of total citations. These findings suggest that while the USA produces the highest volume of publications, countries such as Finland, Switzerland, and Argentina, despite lower output, are generating research of particularly high quality and impact.

### Analysis of authors

**Fig. 6:**
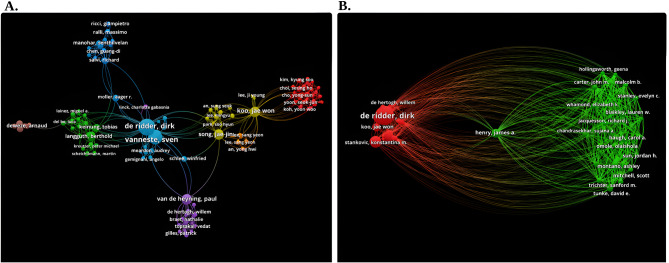
The analysis of authors. (**A**) Network map of co-authors collaboration. The size of the nodes represent the number of publications per author, and the links represent collaborations between authors. (**B**) Network map of bibliographic coupling. Similarly, the size of the nodes represents the number of publications per author, and the links represent bibliographic coupling between authors

VOSviewer was utilized to analyze 130 authors with at least 1 coauthorship and 51 authors with at least 3 publications. The most cited authors are Sujana Chandrasekhar (1158), David Baguley (889), and Deborah Hall (889). The author with the most published papers is Dirk de Ridder (17), followed by Sven Vanneste (14), and Jae-jin Song (9) (Table 1). According to Fig. 6A, there are 11 clusters of authors who frequently collaborate with each other. The red cluster is exceptionally tightly connected, and appears to comprise authors of a single nationality - Korean. Strikingly, James A. Henry appears to be the sole author with strong bibliographic coupling links to both of the extremely distinct clusters in Fig. 6B, suggesting the author’s publications are well-grounded in current literature. It is also noteworthy that James A. Henry is in the top five most cited and most published authors in this research area, indicating a high level of quality research contributions.

### Analysis of institutions

**Fig. 7:**
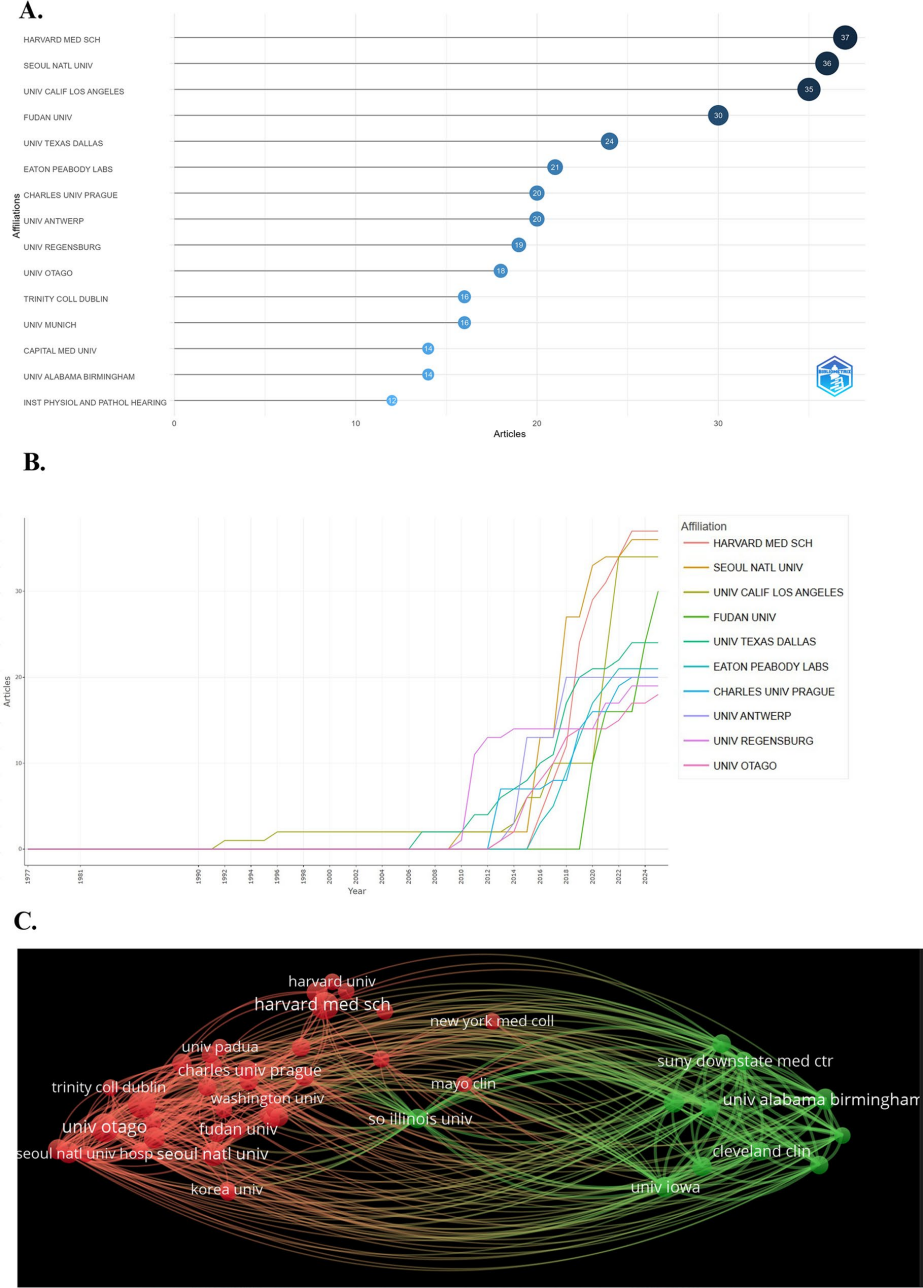
Analysis of institutions. (**A**) Total articles per institution. (**B**) Institutional publication trends over 1977–2025. (**C**) Network analysis of bibliographic coupling between institutions

Figure 7A displays the top 15 research institutions ranked by number of publications in tinnitus pathology, with Harvard Medical School leading with 37 publications, followed closely by Seoul National University (36 publications), and the University of California, Los Angeles (35 publications). This ranking trend is further illustrated in Fig. 7B, which depicts the publication trends of the top 10 institutions. Notably, the three leading institutions rose to prominence around 2014, which corroborates the findings in Section “Analysis of journals” that all journals surged in tinnitus pathology publications around 2014. Interestingly, Southern Illinois University, the Mayo Clinic, and New York Medical College exhibit the highest number of bibliographic coupling links and appear to bridge two distinct clusters of institutions (Fig. 7C). This finding suggests that these three institutions are engaged in research that overlaps thematically with both clusters.

### Analysis of keywords

**Fig. 8:**
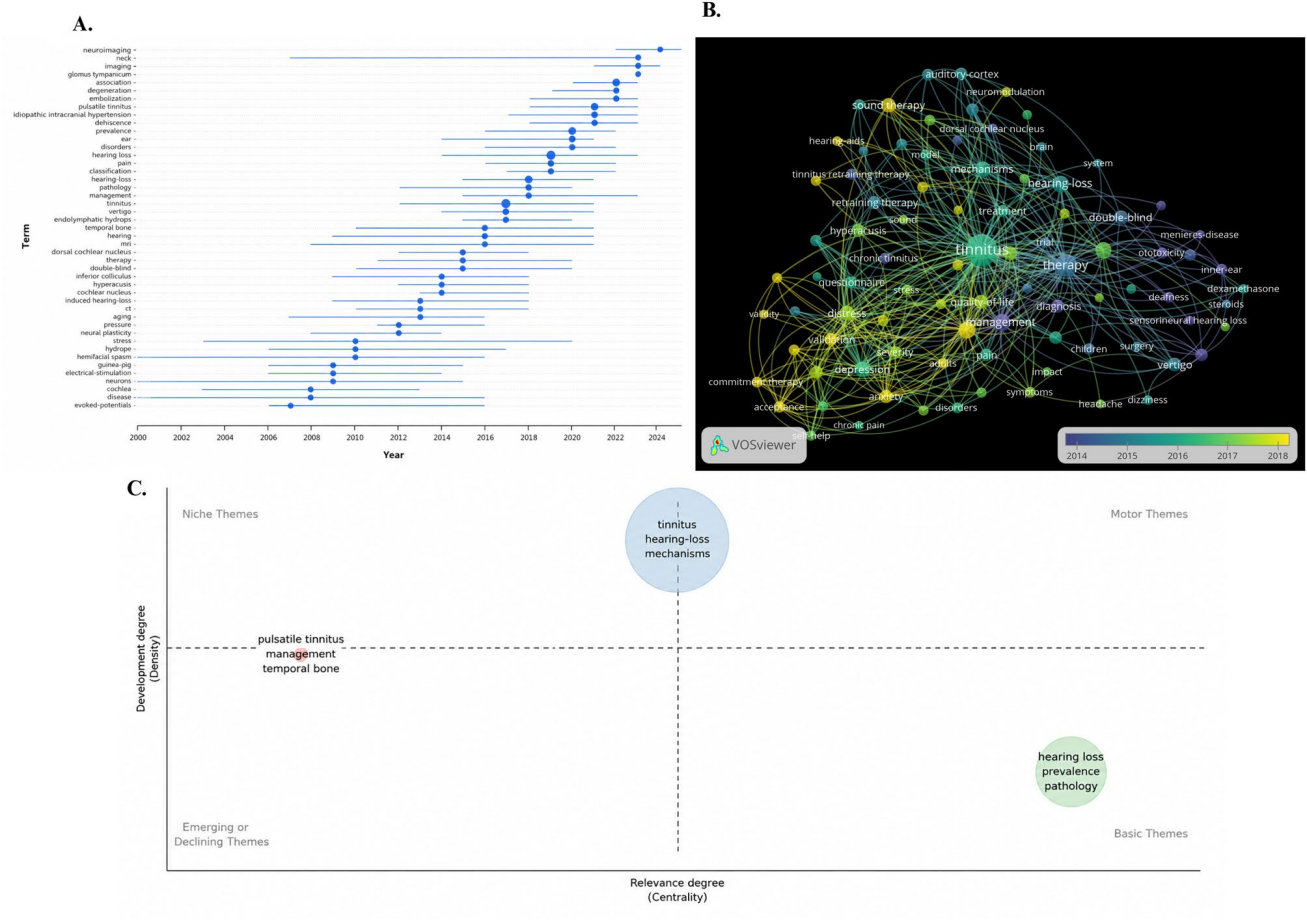
The analysis of keywords. (**A**) Trend topics map over 2000–2025. (**B**) Temporal network analysis of keywords co-occurrence over 2012–2020. (**C**) Thematic map strategic diagram of tinnitus pathology. The centrality (x-axis) “measures for a given cluster the intensity of its links with other clusters.” The density (y-axis) “characterizes the strength of the links that tie the words making up the cluster together.” The motor themes are central and developed, the basic themes are central and underdeveloped, the niche themes are peripheral and developed, and the emerging or declining themes are peripheral and underdeveloped [44]

Keywords are unique words authors believe best represent their papers, and can illuminate current or past trending research topics. During the early years, topics such as “cholesteatoma” (5), “hydrops” (6), “middle-ear” (6), and “vertigo” (36) were the focus of tinnitus pathology research [Fig. 8B]. These early keywords are consistent with views of tinnitus as a peripheral auditory pathology, such as cholesteatoma and Merniere’s disease. The 2015–2020 period marks a significant paradigm shift in tinnitus pathology research from peripheral ear-focused models to a centralized, neurophysiological framework. Keywords such as “auditory cortex” (7), “circadian rhythm” (3), and “mechanisms” (38) reflect a deeper understanding of tinnitus as not just an otologic symptom, but a complex neurological phenomenon. The period 2021–2025 is characterized by a partial return to peripheral ear models and a research focus on interventions, with keywords such as “cochlear synaptopathy” (10), “chronic otitis media” (4), and “surgery” (21) [Fig. 8A and B].

The thematic map diagram displayed in Fig. 8C provides a digestible representation of tinnitus pathology’s research themes and their centrality and development. The blue cluster “tinnitus,” “hearing loss,” and “management” have a large number of publications and have reached maturity. The red cluster (“pulsatile tinnitus,” “management,” and “temporal bone”) is in the process of development, though it is not a main focus of the field. Of most interest here is the green cluster of keywords: themes that are of high centrality to the field yet are underdeveloped and require more academic attention. These themes include “hearing loss,” “prevalence,” and “pathology.”

## Discussion

The present study evaluated 638 publications from 1977 to 2025 on tinnitus pathology research from the WoSCC. Though the field of tinnitus pathology research is relatively small, it has been steadily garnering academic attention for the past five decades and is expected to grow even more rapidly in the future. Journals leading this increase in tinnitus pathology research are Hearing Research, Otology & Neurotology, and Journal of Laryngology & Otology, and leading institutions are Harvard Medical School, Seoul National University, and University of California, Los Angeles. Notably, the high bibliographic coupling of Southern Illinois University, the Mayo Clinic, and New York Medical College with all clusters position the three institutions for future institutional collaborations.

Analysis of publications and collaborations reveals that the leading countries involved in tinnitus pathology research are mainly stationed in North America, Europe, and Asia, while countries such as Iran, Grenada, and Morocco are relatively isolated. These findings support the idea that wealthy, developed countries dominate research in tinnitus pathology.

Regarding individual author contributions, Dirk de Ridder stands out, leading in academic output and H-index. De Ridder famously proposed the thalamocortical dysrhythmia hypothesis, which suggests aberrant network oscillations between the thalamus and cortex underlie phantom sensations [45], and the triple network model, which states that tinnitus occurs when the default mode network, central executive network, and salience network are all active [46]. Additionally, James A. Henry has been identified as a critical bridge in the academic community, particularly between the thematic topics of tinnitus pathology and translational research [47].

Keyword analysis reveals the evolving focus of tinnitus pathology research across different time periods. Early studies emphasized peripheral auditory conditions, 2015–2020 studies shifted toward a neurophysiological framework, and most recent topics focus on intervention. Future directions were highlighted in the thematic map analysis, including “prevalence,” “diagnosis,” and “pathology.” Tinnitus prevalence still suffers significant knowledge gaps, as studies lack standard survey methodology resulting in inconsistent prevalence figures (published figures vary from 5.1 to 42.7%) [6, 48, 49]. Tinnitus pathology is infamously heterogeneous and there is a dire need for specific diagnosis and personalized treatment [50, 51, 1]. 

From an emergency medicine perspective, tinnitus represents a diagnostically challenging symptom because it may reflect either a benign chronic condition or a manifestation of serious neurologic, vascular, or otologic disease. Emergency physicians frequently encounter patients presenting with acute tinnitus in conjunction with sudden sensorineural hearing loss, vertigo, focal neurologic deficits including posterior circulation stroke, or vascular tinnitus. The evolution of tinnitus pathology research toward neurophysiological and network-based models may ultimately contribute to improved diagnostic frameworks and risk stratification strategies in acute-care settings. Continued investigation into tinnitus mechanisms therefore has implications not only for specialty care but also for emergency evaluation and management.

## Limitations

The most important limitation lies in the fact that data was only procured from the WoSCC. This results in potential biases in journal selection, language limitation (as the data is primarily English), and exclusion of non-journal publications such as books and conference papers [36]. Future research could benefit from sourcing data from other important databases such as Google Scholar, PubMed, and Scopus to enrich the sample and increase generalizability of results. Despite these limitations, bibliometric analysis still offers valuable insights into the field of tinnitus pathology.

## Conclusions

The bibliometric analysis of tinnitus pathology has uncovered valuable insights into the current landscape and developmental trajectory of this promising field. Tinnitus pathology research, though relatively niche, has steadily grown and is expected to continue to expand. Research leadership is concentrated in developed regions, with top institutions and journals driving the bulk of academic output. Thematic evolution in keyword analysis found the field shifted from peripheral to central nervous mechanisms, with recent attention on translating findings to intervention. Despite progress, significant gaps remain in standardized prevalence data, diagnostic specificity, and the development of personalized treatment approaches to promote more effective tinnitus management.

## Data Availability

No datasets were generated or analysed during the current study.
